# Presidential Symposium: Looking Over the Horizon: States as the Laboratory for Policy Innovations

**DOI:** 10.1093/geroni/igaf122.1659

**Published:** 2025-12-31

**Authors:** Howard Degenholtz

## Abstract

Supreme Court Justice Louis Brandeis suggested that states are the laboratories of democracy. In a year in which so much attention is at the federal level, this year we will focus on the ways in which we turn our gaze to the states as sources to learn about innovations in policies that support older adults. States have long been the incubators for new ideas that have had dramatic impact. For example, the notion of using Medicaid dollars to provide long-term services and supports in people’s own homes rather than in institutional settings had its start through state demonstrations. This symposium will feature innovative state programs that support caregivers for people with dementia, the creation of a long-term care insurance program, and models for paying family caregivers. Speakers will describe how states have created these programs and learned from each other, notable accomplishments and challenges in implementing them, and what they expect to see moving forward. They will also touch on where and how researchers can play an important role in studying and advancing state-level innovations.

